# Non-pharmacological approaches for migraine management: a mini-review

**DOI:** 10.3389/fpain.2026.1760756

**Published:** 2026-03-09

**Authors:** Vineeta Singh, Anand Kumar, Sucharita Ray, Kamalesh Chakravarty, Neha Lall, Deepika Joshi

**Affiliations:** 1 Institute of Medical Sciences, Banaras Hindu University, Varanasi, India; 2 Post Graduate Institute of Medical Education and Research, Chandigarh, India; 3 Indira Gandhi Institute of Medical Sciences, Patna, India

**Keywords:** acupuncture, cognitive behavioural therapy, cortical spreading depression, exercise, manual therapy, migraine, mindfulness

## Abstract

**Background:**

Migraine is a neurological condition resulting from intricate connections among cerebral excitability, the nervous system, muscular function, and the body's stress response. For various reasons, complementary and non-pharmacological interventions were explored for the management of migraine.

**Methods:**

We conducted a targeted review of existing research using PubMed. Our search focused on studies that use various non-drug approaches, such as yoga, acupuncture, manual therapy, exercise, and behavioural therapies (e.g., biofeedback, cognitive behavioural therapy), that affect migraine sufferers. Our main goals were to determine whether these methods reduced the frequency of migraines, their duration, or the severity of pain, and to assess any adverse side effects based on high-quality studies, such as randomised controlled trials.

**Results:**

The studies we examined consistently showed that these non-drug interventions significantly cut down on how often people get migraines, how severe they are, and how much they disrupt daily life. Practices like yoga improve the autonomic stability, while acupuncture provided lasting relief from headache days. Regular exercise and manual therapy helped reduce muscle tension and headache frequency. Behavioural techniques, such as relaxation training and biofeedback, were particularly effective at helping patients cope with stress and improve their overall function. Importantly, these treatments had very few negative side effects.

**Conclusion:**

Evidence from controlled studies indicates that traditional, behavioral, and manual non-pharmacological therapies offer effective, well-tolerated options for migraine management. These therapies may provide a vital alternative or addition to medication, helping us move toward a more personalised and holistic approach to migraine care.

## 1 Introduction

Migraine is considered one of the most prevalent neurological disorders worldwide, affecting approximately 15% of the global population and contributing substantially to global disability, particularly among women of reproductive age (1, 2). An Indian hospital-based study also demonstrates a high burden and distinct clinical patterns of primary headaches, including migraine (3). Along with severe episodic pain, migraine exhibits a broad spectrum of sensory, cognitive, and autonomic symptoms, as photophobia, phonophobia, nausea, vomiting, dizziness, and mental fog, which affect productivity and quality of life (4). Despite advances in modern pharmacotherapy, including triptans, gepants, ditans, beta-blockers, anti-epileptics, antidepressants, and monoclonal antibodies targeting calcitonin gene-related peptide (CGRP), many individuals experience insufficient efficacy, intolerable side effects, high cost, or contraindications (5, 6). Moreover, medication-overuse headache remains a significant challenge in long-term management.

Migraine is increasingly understood as a complex neurobiological disorder involving altered cortical excitability, metabolic vulnerability, and neuroimmune mechanisms. Given that migraine is a multisystem disorder, there is increasing interest in safe and effective consultation. At the same time, individuals with chronic or refractory migraine frequently require multimodal approaches that extend beyond pharmaceuticals (7). Therefore, this review summarises current evidence on non-pharmacological interventions, including neuromodulation, acupuncture, psychotherapy, physiotherapy, exercise, and integrative therapies to highlight their clinical relevance and potential role in contemporary migraine management. Many patients remain reluctant to use or continue preventive medications due to variable efficacy, adverse effects, or personal preference, and a substantial proportion seek non-pharmacological therapies.

## 2 Method

This mini-review used a systematic PICO-based methodology. Population: adults and adolescents diagnosed with migraine, with or without aura. Interventions: non-pharmacological and complementary-alternative therapies, encompassing yoga, pranayama, meditation, mindfulness, Ayurvedic treatments, acupuncture, biofeedback, cognitive behavioral therapy (CBT), relaxation methods, physical therapy, and organized exercise. Comparators: placebo or sham interventions, standard medical treatment, wait-list controls, or alternative non-pharmacological therapies. Outcomes: frequency of migraines, number of headache days, pain intensity, duration of attacks, medication usage, disability scores, quality of life, psychological well-being, sleep quality, and adverse events.

A PubMed search was performed utilizing specified MeSH terms and phrases related to migraine, alternative therapies, yoga, Ayurveda, acupuncture, behavioral therapy, and physical rehabilitation. This search showed 3,107 records. Following an initial screening of titles and abstracts for relevance, 984 articles were retained based on the availability of free full text. Further, a final set of 59 core references was selected for the present study. Eligible research included randomised controlled trials, controlled clinical trials, and quasi-experimental methods.

## 3 Non-Invasive neuromodulation

Non-invasive neuromodulation has gained considerable attention as a safe and well-tolerated therapeutic option for migraine, particularly for patients who prefer to avoid pharmacological treatments or experience suboptimal response to medication. These techniques modulate cortical excitability, trigeminovascular activity, or brainstem autonomic pathways through externally applied electrical or magnetic stimulation. A growing body of evidence supports their utility in both acute treatment and preventive therapy (8).

Supraorbital transcutaneous nerve stimulation, exemplified by the Cefaly device, is one of the most widely studied modalities. Randomised controlled trials demonstrate modest but meaningful reductions in monthly migraine days and higher responder rates compared with sham stimulation, with paraesthesia being the most commonly reported but reversible adverse event. Similarly, transcutaneous occipital nerve stimulation has shown beneficial effects on headache frequency, particularly with higher-frequency stimulation parameters. However, clinical evidence remains limited by small sample sizes and potential carryover effects (9).

Non-invasive vagus nerve stimulation using a handheld cervical device (gammaCore) has demonstrated feasibility and good tolerability in both acute and preventive settings. Open-label studies report notable pain freedom at 2 h, and menstrual-related migraine appears particularly responsive, however, randomized trials in chronic migraine yield mixed results, indicating that more rigorous evaluation is needed to clarify its preventive efficacy (10).

Single-pulse transcranial magnetic stimulation (sTMS) is supported by randomized trials showing significant improvements in 2-hour pain freedom for acute treatment of migraine with aura. Longer-term post-marketing data reveal reductions in headache days in both episodic and chronic migraine with consistent daily use. The favourable safety profile and at-home usability of sTMS make it a compelling option for patients seeking non-drug-based prevention (11).

Other emerging modalities include transcranial direct current stimulation, which may modulate cortical excitability through anodal or cathodal stimulation, and remote electrical neuromodulation, a smartphone-controlled arm-based stimulation technique that leverages conditioned pain modulation to abort acute attacks. While early studies show promising signals of efficacy, larger and longer-term trials are still required (12, 13).

## 4 Invasive neuromodulation

Invasive techniques comprising stimulation of the occipital nerve, sphenopalatine ganglion, or cervical spinal cord may be considered for chronic migraine patients who have consistently failed prior treatments. These approaches involve surgical implantation of electrodes to modulate pain pathways within the tri-geminocervical system and are considered only when non-invasive and pharmacological interventions have been exhausted. Recent long-term data on paraesthesia-free high-frequency (10 kHz) cervical spinal cord stimulator provide some evidence of meaningful benefit in refractory cases. Although such findings are promising, the invasive nature, higher procedural risk, and cost of implanted neuro-modulation systems limit their use to carefully selected refractory patients, for whom they may offer a valuable but last-line therapeutic option (14).

## 5 Acupuncture and related needling therapies

### 5.1 Traditional acupuncture

Acupuncture is one of the most extensively studied non-pharmacological interventions for migraine, with several high-quality randomised trials and meta-analyses supporting its efficacy. Among the most influential contributions is the individual patient data meta-analysis by Vickers et al. (2018), which showed data from 20,827 patients across 39 trials, the most extensive and most rigorous data set available for acupuncture research (15). This analysis demonstrated that acupuncture was significantly superior to both sham acupuncture and no-acupuncture controls for chronic pain conditions. One of them is chronic headache, a finding that aligns with earlier Cochrane-level evidence showing that true acupuncture is more effective than sham for migraine prophylaxis (16). The effect sizes were moderate, clinically meaningful, and importantly, persistent over time, with only a 15% reduction in treatment benefit at one-year follow-up, indicating durable efficacy (15).

Moreover, evidence specific to migraine is strengthened by a multicentre randomised clinical trial by Xu et al. (2020) involving 150 acupuncture-naïve patients with episodic migraine without aura (16). Although most reviews were rated as low or critically low quality using AMSTAR-2, the GRADE assessment identified high-quality evidence indicating that acupuncture was superior to Western medicine for reducing headache frequency, headache days, headache attack intensity, and medication use (17).

Acupuncture also outperformed sham acupuncture in several studies, suggesting therapeutic effects beyond placebo mechanisms (18, 19). According to Zhao et al. (2017), acupuncture established its superiority over both sham and topiramate (17), while Li et al. (2020) provided compelling proof of acupuncture's benefits over Western treatment, emphasizing its few side effects (18).

#### 5.1.1 Auricular acupuncture

Auricular acupuncture has received comparatively less attention. A study by Zhang et al. (2020) highlights the need for well-designed randomised trials to evaluate the therapeutic potential for migraine (20). This approach offers convenience advantages and might work by the modulation of cranial nerves and core pain networks.

### 5.2 Dry needling and trigger point therapy

Dry needling represents a distinct needling approach based on Western anatomical and neurophysiological principles rather than traditional meridian theory. In a systematic review of 8 randomised clinical trials, Vázquez-Justes et al. (2022) showed that dry needling significantly improves sensory and functional outcomes across multiple headache types, including migraine (21). This aligns with the broader musculoskeletal pain literature, which shows that dry needling can decrease nociceptive input, reduce myofascial trigger point activity, and modulate central sensitisation processes. The therapeutic mechanism differs from traditional acupuncture as the dry needling specifically targets pericranial and cervical myofascial trigger points that contribute to migraine pathophysiology. Common target areas include the suboccipital muscles, upper trapezius, sternocleidomastoid, and temporalis muscles. The technique aims to elicit a local twitch response, releasing muscle contracture and improving local blood flow.

While both acupuncture and dry needling involve needle insertion, clinicians should recognize the fundamental difference in assessment and treatment planning as the acupuncture follows meridian-based diagnosis, while dry needling relies on identification of specific trigger points through palpation and assessment of referred pain patterns.

## 6 Physical and behavioural interventions

Physical and behavioral therapies represent crucial non-pharmacological approaches targeting musculoskeletal, metabolic, neurophysiological, and psychological mechanisms often poorly responsive to pharmacological interventions alone. These interventions address cervical dysfunction, decreased aerobic fitness, autonomic dysregulation, stress sensitivity, and maladaptive cognitive patterns.

### 6.1 Part-A physical interventions

#### 6.1.1 Exercise-based physical therapy

Physical therapy is mainly beneficial for cervical musculoskeletal dysfunction, postural abnormalities, and impaired sensorimotor control (5, 22). A 2023 systematic review showed that aerobic exercise in combination with medication decreases the headache frequency, duration, and intensity (23).

Physical therapy programs mainly include cervical spine stabilisation exercises, postural retraining (especially for forward head posture), Vestibular rehabilitation for balance impairment, therapeutic exercise for strength and flexibility and patient education on ergonomics and self-management.

#### 6.1.2 Manual therapy (integrated approaches)

Manual therapies are hands-on techniques that are used across multiple healthcare professions to report musculoskeletal dysfunction, pain, and movement impairment. However, recent IASP Task Force guidelines on integrated manual therapies, contemporary evidence-based practice emphasizes multimodal approaches combining various techniques (joint mobilization/manipulation, soft tissue therapy, myofascial release, neural mobilisation, muscle energy techniques) with active interventions rather than isolated passive treatments (24, 25).

Manual therapy demonstrates effectiveness comparable to prophylactic medications (propranolol, topiramate) for migraine prevention (1), with systematic reviews showing significant improvements in disability, quality of life, and pain outcomes (26). A 2024 meta-analysis reported, large effect sizes for cervicogenic headache (SMD −2.01) and moderate effects for tension-type headache (SMD −0.86), with variable effects in migraine depending on the presence of cervical dysfunction and myofascial trigger points (27).

These interventions work via multiple mechanisms modulating pain through neurophysiological pathways, addressing biomechanical dysfunction in the cervical and cranial regions, reducing myofascial trigger point activity, improving fascial mobility, and influencing central pain processing. Optimal outcomes are achieved when manual techniques are integrated with therapeutic exercise programs and patient education, addressing both passive tissue restrictions and active movement dysfunction (1, 5, 24–27).

### 6.2 Part B: behavioral interventions

#### 6.2.1 Cognitive behavioural therapy

CBT addresses maladaptive beliefs and stress responses that alter cortical excitability and lower attack thresholds. A meta-analysis of 11 Randomized control trial (RCTs) demonstrated CBT reduces headache frequency and MIDAS ratings (28), proving especially beneficial for chronic and medication-overuse migraines by mitigating stress responses, regulating autonomic balance, and reducing anxiety and depression (29, 30).

CBT for migraine typically includes identifying and modifying pain-related catastrophizing, challenging beliefs about headache control, developing adaptive coping strategies, behavioral activation for comorbid depression, and establishing sleep hygiene and routine. These components work synergistically to reduce the psychological burden of migraine, improve patients' sense of control over their condition, and address common comorbidities that can exacerbate headache frequency and severity.

#### 6.2.2 Mindfulness-based stress reduction (MBSR)

MBSR is centred on the importance of being aware of the present moment without judgment and of being able to ride through difficult emotions. An RCT that lasted 36 weeks found that MBSR significantly lowered disability, pain catastrophizing, sadness, and the unpleasantness of pain (31). Mindfulness makes stress responses better (32). This is especially helpful for people who are easily stressed, as it improves heart rate variability and autonomic stability (33).

#### 6.2.3 Biofeedback and relaxation therapies

Biofeedback provides real-time physiological data (muscle tension, skin temperature, heart rate) enabling voluntary control over autonomic functions. Systematic reviews report positive outcomes in anxiety reduction and autonomic regulation (34). Relaxation training (diaphragmatic breathing, progressive muscle relaxation, guided imagery) downregulates stress responses and sympathetic overactivity (35). These therapies reduce peri-cranial muscle tension, improve vagal tone, and lower stress-related cortisol levels, making them especially useful for stress-triggered attacks.

#### 6.2.4 Multicomponent behavioral programs

Combining behavioral strategies yields more substantial benefits. A 2025 systematic review of 50 adult RCTs found CBT, relaxation training, and mindfulness each reduced migraine frequency, while behavioral education improved disability outcomes (36). Integrated programs combining CBT, biofeedback, and relaxation demonstrate powerful results across age groups (37).

The recent work by Mínguez-Olaondo et al. (2024) emphasizes the expanding therapeutic arsenal of behavioral approaches and their critical role in comprehensive migraine management, particularly for patients with significant psychological comorbidities or medication limitations (38).

## 7 Yoga and traditional medicine approaches

Traditional medical systems, including yoga, acupuncture, and herbal therapies, are increasingly recognised for migraine management, addressing physical, psychological, and energetic aspects through mind-body integration and lifestyle modification.

### 7.1 Yoga and meditation

Yoga combines breathing, meditation, and postures to provide mind-body benefits. A 2020 meta-analysis demonstrated significant reductions in headache frequency, pain intensity, and duration, with notable effects for tension-type headaches and moderate benefits for migraine (39, 40). Kumar et al., (2020) in CONTAIN RCT, involving 160 adults with episodic migraine were compared standard medical therapy with structured yoga program. After 3 months, the yoga group showed significantly greater reductions in headache frequency (*Δ*3.53), intensity, HIT-6 scores, MIDAS disability, and pill consumption than the control group, demonstrating the superiority of yoga as an add on treatment (41). Mechanisms include improved autonomic modulation, reduced muscle tension, enhanced respiratory efficiency, and stress reduction through hypothalamic–pituitary–adrenal (HPA) axis regulation (42, 43).

### 7.2 Traditional Chinese medicine (TCM)

TCM herbal formulations show promise for migraine prevention. Chinese patent medications such as Yangxue Qingnao granules and Toutongning capsules aim to regulate body temperature and enhance blood flow (44). RCTs have demonstrated that oral TCM formulations significantly outperform controls in vestibular migraine, reducing the frequency and duration of vertigo (17, 45, 46).

### 7.3 Ayurvedic medicine

Ayurveda regards migraines (Ardhavabhedaka) as Tridoshaja disorders associated with Pitta and Vata doshas, emphasizing the restoration of doshic balance through detoxification, herbal treatments, dietary modifications, and lifestyle changes (47). A narrative analysis found 12 clinical studies (2000–2020), 11 of which showed therapeutic benefit as monotherapy and no significant side events were observed (48, 49). A comprehensive observational study involving 406 individuals indicated that 70.5% saw significant reductions in migraine frequency and pain severity after adhering to a standardized 90-day Ayurvedic program (49).

Key bio-purification procedures in Panchakarma therapies are: (i) Nasya (nasal therapy with medicated oils) modulating trigeminal nerve activity and autonomic balance (51) (ii) Virechana (therapeutic purgation) eliminating excess Pitta and addressing gut-migraine associations (51) (iii) Shirodhara (oil dripping therapy) calming the mind and modulating cortical excitability (52) and (iv) Siravyadha (therapeutic bloodletting) for blood-impurity related migraines with dramatic MIDAS score improvements (48).

Pathyadi Kwatha, Shirashooladi Vajra Rasa, Sootshekhar Rasa, and Avipattikara Churna are traditional medicines for migraines that are usually induced by Pitta (48–50, 53). Further, some of the important medicinal plants are Bacopa monnieri, which protects the brain and improves blood flow to the brain (54), Withania somnifera, which reduces inflammation in the brain and affects the HPA axis (55, 56), Zingiber officinale, which has been shown in clinical studies to relieve migraines as well as sumatriptan (57, 58), Terminalia chebula, which has antioxidant and neuroprotective properties (59, 60), and Curcuma longa, which reduces inflammation and may block CGRP-related pathways (61).

## 8 Discussion

The findings of this comprehensive review demonstrate that non-pharmacological and traditional medicine interventions including acupuncture, exercise and physical therapy, behavioral strategies, yoga, TCM, and Ayurvedic approaches offer substantial therapeutic value in the prevention and management of migraine. These modalities act through neurophysiological, metabolic, musculoskeletal, autonomic, and psychological mechanisms that complement or enhance conventional pharmacotherapy (15, 49) (Table 1).

**Table 1:** Evidence summary and clinical application guide for Non-pharmacological migraine interventions.

Intervention	Evidence & Size	Quality of Evidence	For Clinical Indications	References
Acupuncture	High: Moderate-large effect on frequency and intensity; sustained benefit (15% reduction at 1 year)	High (IPD meta-analysis 20,827 patients; multiple large RCTs)	• Medication intolerant or inadequate response • Seeking evidence-based non-pharmacological prophylaxis • Long-term sustainable option • Pregnant/breastfeeding women	(15–19)
Auricular Acupuncture	Low-Moderate: Positive results in previous RCTs; convenience advantages	Low (limited high-quality trials)	• Long-term migraine management • Convenience/patient comfort priority • Adjunct to body acupuncture	(20)
Dry Needling	Moderate: Significant functional improvement, comparable to traditional PT	Low-Moderate (8 RCTs, heterogeneous quality)	• Myofascial trigger points (pericranial, cervical) • Persistent neck/shoulder tension • Adjunct to physical therapy	(21)
Strength Training	Moderate-High: MD −3.55 monthly migraine days (exceeds topiramate/amitriptyline)	Moderate (network meta-analysis, 21 trials, 1,195 patients)	• Sedentary lifestyle, low physical fitness • Cervical/shoulder muscle weakness • Metabolic syndrome • Preference for self-managed intervention	(23)
High-Intensity Aerobic Exercise	Moderate-High: MD −3.13 monthly migraine days	Moderate (network meta-analysis)	• Low aerobic fitness • Metabolic syndrome, obesity • Comorbid depression/anxiety • Cost-effective long-term option	(23, 25)
Moderate Aerobic Exercise	Moderate: MD −2.18 monthly migraine days	Moderate	• Initiating exercise program (safer entry point) • Elderly patients • Cardiovascular considerations	(23, 25)
Manual Therapy (Integrated Approaches)	Moderate-High: Comparable to propranolol/topiramate for prophylaxis. Large effect for cervicogenic headache (SMD −2.01), moderate for TTH (SMD −0.86), variable for migraine. Significant improvement in disability, quality of life, and pain outcomes when combined with exercise	Moderate (systematic reviews, meta-analyses, IASP Task Force guidelines)	• Cervical dysfunction, neck pain, forward head posture • Myofascial trigger points (suboccipital/trapezius) • Fascial restriction and muscle stiffness • Temporomandibular dysfunction • Vestibular impairment/imbalance • Comorbid cervicogenic or tension-type headache • Preference for hands-on approaches • Best outcomes when combined with exercise therapy	(1, 5, 24–27)
Cognitive Behavioral Therapy	Moderate-High: Significant reduction in frequency, MIDAS scores, and headache intensity	Moderate (meta-analysis, 11 RCTs)	• High stress, anxiety, sleep disturbances • Medication-overuse headache • Comorbid depression/anxiety • Significant disability despite frequency control • Adolescents	(28–30)
Mindfulness-Based Stress Reduction	Moderate-High: Similar frequency reduction to education, superior improvement in disability, quality of life, catastrophizing, depression	Moderate-High (large RCT with 36-week follow-up)	• High stress sensitivity • Poor sleep quality • Emotional dysregulation • Pain catastrophizing • Preference for mind-body approaches	(33)
Biofeedback	Moderate: Moderate effect on frequency; improved autonomic regulation	Moderate (systematic reviews, multiple RCTs)	• Muscle tension-related migraine • High anxiety • Stress-triggered attacks • Poor autonomic control • Pediatric/adolescent populations	(34, 35)
Relaxation Training	Moderate: Moderate reduction in frequency and intensity	Moderate (multiple RCTs)	• Stress-driven migraine • Pericranial muscle tension • Combination with biofeedback or CBT • Cost-effective option	(35, 36)
Yoga	Moderate: Significant reduction in frequency, duration, and pain intensity; particularly strong for tension-type headache	Moderate (meta-analysis of RCTs)	• Stress-exacerbated migraine • Autonomic dysregulation • Comorbid anxiety/depression • Preference for integrative mind-body practice • Poor posture/flexibility	(39–42)
Meditation/Mindfulness Practices	Moderate: Indirect frequency reduction via improved sleep; improved emotional regulation	Moderate (RCTs)	• Sleep disturbances • High stress reactivity • Emotional triggers • Circadian dysregulation	(51, 52)
Traditional Chinese Medicine (Herbal)	Low-Moderate: Higher effectiveness rates vs. controls in limited trials	Low-Moderate (heterogeneous trials, standardization issues)	• Vestibular migraine symptoms • Preference for holistic/traditional approaches • Cultural congruence • Limited access to conventional care	(44–46)
Ayurveda: Panchakarma+Herbal	Moderate: 35.2% complete resolution	Low-Moderate 5RCT	• Preference for natural/holistic approaches • Digestive comorbidities (IBS, reflux/hyperacidity) • Cultural congruence (South Asian populations) • Medication intolerance • Stress-exacerbated migraine • Seeking detoxification-based approaches	(48–50)
Ayurveda: Specific Procedures	Nasya: RCT showing significant severity reduction Virechana: Part of multi-intervention protocols Shirodhara: Case reports/observational	Low-Moderate (limited RCTs for individual procedures)	• Nasya: Supraclavicular region disorders, Vata-Kapha imbalance • Virechana: Pitta-predominant migraine, digestive issues • Shirodhara: High stress, anxiety, insomnia • Siravyadha: Rakta Dushti signs (photophobia, burning, throbbing)	(46, 49–53
Ayurveda: Key Herbs	Variable: Strong mechanistic basis; clinical evidence varies by herb	Low-Moderate (mostly preclinical+some clinical trials)	• Ashwagandha: Stress-induced migraine, anxiety, HPAdysregulation • Brahmi: Cognitive fog, poor concentration • Ginger: Acute migraine+nausea (comparable to sumatriptan in RCT) • Turmeric: Inflammatory-type migraine • Haritaki: Oxidative stress, neuroprotection needs	(54–61)

Quality of Evidence: High (large meta-analyses/multiple RCTs), Moderate (systematic reviews with limitations), Low-Moderate (limited/heterogeneous trials).

Effect Sizes: MD = Mean Difference in monthly migraine days (≥2 days=clinically significant); SMD=Standardized Mean Difference (0.2–0.5 small, 0.5–0.8 moderate, >0.8 large).

Clinical Practitioners: Acupuncture (acupuncturists/TCM practitioners), Dry Needling (physiotherapists), Exercise Therapy (physiotherapists/exercise specialists), Manual Therapy (osteopathic physicians, physiotherapists, chiropractors, manual therapists), Physical Therapy (physiotherapists), CBT (psychologists/therapists), MBSR (mindfulness instructors), Biofeedback (psychologists/specialists), Yoga (yoga instructors/therapists), TCM Herbs (TCM practitioners), Ayurveda (Ayurvedic practitioners).

Clinical Reasoning Frameworks: Each intervention employs distinct assessment and treatment approaches. Acupuncture uses meridian theory, dry needling targets myofascial trigger points, manual therapy integrates biochemical, neurophysiological, and pain science perspectives, physical therapy focuses on biomechanics and exercise, behavioral therapies target psychological and autonomic mechanisms, traditional medicines use holistic constitutional models.

Manual Therapy Note: Per IASP Task Force guidelines (24), manual therapy is an integrated multimodal approach used across multiple professions, not profession-specific. Optimal practice combines various manual techniques with active interventions (exercise, education). Effectiveness depends on technique integration and clinical reasoning rather than professional discipline.

### 8.1 Comparative effectiveness across modalities

Acupuncture demonstrates the strongest and most consistent evidence. In comparison to sham and no-acupuncture controls, the Individual Participant Data meta-analysis by Vickers et al. (2018) included 20,827 patients and showed significant benefits for chronic headaches, with a 15% reduction in effectiveness at one year (15). According to Xu et al. (2020), manual acupuncture reduced migraine days more successfully than sham (16). Further, Li et al. (2020) provided compelling proof of acupuncture's benefits over Western treatment, emphasizing its few side effects (18), while Zhao et al. (2017) established its superiority over sham and topiramate (17).

Exercise and physical therapy are strongly supported interventions. Woldeamanuel and Oliveira (2022) (23) reported that strength training results in a higher reduction in migraine frequency, followed by high-intensity and moderate-intensity aerobic exercise. While manual treatment is as effective as propranolol or topiramate, physical therapy and multimodal programs provide broader clinical benefits (1, 27). These interventions likely facilitate improvement via musculoskeletal adjustments, neurophysiological modulation, metabolic augmentation, and autonomic regulation (33).

Behavioural and mind-body therapies address psychological and autonomic pathways inadequately targeted by pharmacotherapy. In addition, CBT, MBSR, relaxation training, and biofeedback consistently reduce disability, improve coping, decrease catastrophizing, and ameliorate comorbid anxiety and depression (30–35). Further, MBSR significantly improved disability, quality of life, catastrophizing, and depression compared to education, despite similar migraine day reductions (33, 41). These interventions modify cognitive, emotional, and autonomic responses to pain, acting through stress regulation, HPA axis modulation, autonomic stabilization, and enhanced pain modulation pathways (29, 32).

### 8.2 Traditional medicine approaches: holistic and culturally congruent options

Notable advantages of yoga and meditation include greater stress management, less muscle tension, increased respiratory efficiency, and improved autonomic regulation. Further, meta-analyses show moderate benefits for migraines and considerable reductions in headache frequency, duration, and intensity, especially for tension-type headaches (39, 40). Additionally, both techniques improve stress pathways and stabilise HPA axis function while reducing anxiety, depression, and sleep disturbances, all typical migraine comorbidities. MBSR has demonstrated the effectiveness of meditation-based therapies improves sleep quality and reduces migraine days (33).

**Figure 1:**
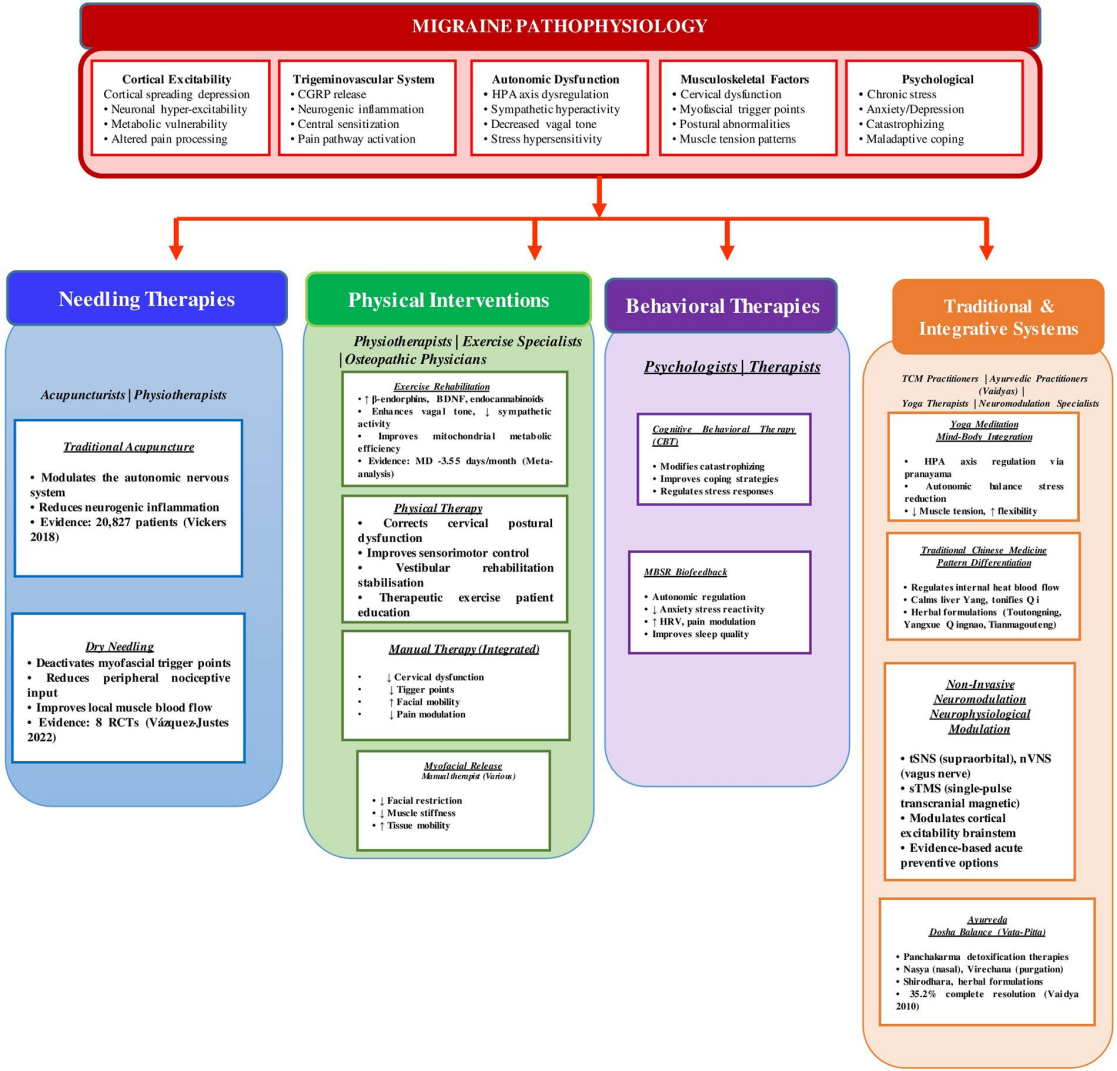
Non-Pharmacological interventions for migraine: An integrative framework.

Herbal remedies such as Toutongning, Yangxue Qingnao, and Tianmagouteng granules are used in TCM to improve blood circulation, and reduce internal heat, and calm the spirit to prevent migraines (45). When compared to controls, RCT using TCM for vestibular migraine shown increased efficacy and decreased vertigo frequency (46). By affecting core pain networks and activating cranial nerves, auricular acupuncture also offers long-term migraine treatment alternatives (47). According to Ayurvedic medicine, imbalances in the Vata and Pitta doshas can cause migraines. Twelve studies conducted between 2000 and 2020 provide evidence that monotherapy is beneficial, with 35.2% of patients experiencing total symptom relief (50). Trigeminovascular modulation and detoxification are the main goals of panchakarma treatments like Nasya, Virechana, and Shirodhara (51, 52). Ayurvedic herbs with migraine-related neuroprotective and anti-inflammatory qualities include Bacopa monnieri and Curcuma longa (61) (Figure 1).

### 8.3 Limitations and future directions

Even though there are promising evidence, non-pharmacological migraine treatments have methodological light, such as small sample sizes, short follow-ups, inconsistent intervention protocols, and blinding issues. Future research should focus on large multi-center RCTs with standardized protocols, reliable outcome, and longer follow-up periods to see if the therapeutic benefits last. Comparative efficacy studies that compare non-pharmacological therapies to current pharmaceutical treatments, as well as personalized medicine strategies that determine which patients respond best to certain treatments, are important for moving the field forward and improving individualized migraine management.

## 9 Conclusion

Non-pharmacological and traditional medicine therapies might substantially improves the migraine prevention and management through the regulation of neurovascular functions, diminishing central sensitisation, rectifying musculoskeletal disorders, and re-establishing autonomic balance. These interventions should be a key part of patient-centred care, ensuring safety, accessibility, and cultural adaptability while addressing the comorbidities. Combining these approaches with pharmacological treatment tailored to individual profiles might optimise the outcomes for migraine patients.
